# Justification of principles for healthcare priority setting: the relevance and roles of empirical studies exploring public values

**DOI:** 10.1136/jme-2022-108702

**Published:** 2023-02-22

**Authors:** Erik Gustavsson, Lars Lindblom

**Affiliations:** 1 Division of Philosophy and Applied Ethics, Department of Culture and Society, Linköping University, Linköping, Sweden; 2 The National Centre for Priorities in Health, Department of Health, Medicine and Caring Sciences, Linköping University, Linköping, Sweden

**Keywords:** Ethics- Medical, Policy, Resource Allocation

## Abstract

How should scarce healthcare resources be distributed? This is a contentious issue that became especially pressing during the pandemic. It is often emphasised that studies exploring public views about this question provide valuable input to the issue of healthcare priority setting. While there has been a vast number of such studies it is rarely articulated, more specifically, what the results from these studies would mean for the justification of principles for priority setting. On the one hand, it seems unreasonable that public values would straightforwardly decide the ethical question of how resources should be distributed. On the other hand, in a democratic society, it seems equally unreasonable that they would be considered irrelevant for this question. In this paper we draw on the notion of reflective equilibrium and discuss the relevance and roles that empirical studies may plausibly have for justification in priority setting ethics. We develop a framework for analysing how different kinds of empirical results may have different kinds of implications for justification.

## Introduction

 The pandemic situation put pressure on questions about how scarce healthcare resources should be distributed. For example, many healthcare systems developed triage guidelines to manage shortages of ventilators.^1^ There has also been an increased interest among researchers to explore public views about these questions.^2^,^4^ In fact, over the last 20 years, there have been numerous studies of public values about the appropriate grounds for healthcare priority setting.^5^,^12^ It is often assumed that such empirical studies provide important input to the discussion about which principles should guide priority setting. However, it remains unclear what role these studies should play, more specifically, and more importantly, what implications these findings would have for the justification of principles for priority setting.

On the one hand, it seems unreasonable that public values would be considered decisive in deciding the *ethical* question of which principles should guide priority setting. The mere fact that some views are supported by public values does not necessarily say anything about their moral rightness.^13^ The social support for, for instance, racist or sexist policies seems to have little (or no) relevance for the question about to what extent such policies are morally justified. This view lends support from philosophically principled accounts about the distribution of goods. On the other hand, it seems equally unreasonable that they would be considered irrelevant in this respect. For instance, the fact that some normative standpoints have implications that are counterintuitive to many people may be taken as a reason to revise these standpoints.^14^,^17^ Accordingly, empirical studies reasonably play *some* role for the justification of principles for priority setting.^1^ 18,23

Drawing on the notion of reflective equilibrium^24^,^28^ we discuss the relevance and roles that empirical studies may plausibly have for the justification of principles for priority setting. The aim for this paper is to develop a framework that can articulate these different roles in relation to empirical studies of public values and make explicit how different empirical results may have different implications for justification.

The article proceeds as follows. In section 2, we will give some preliminaries for the project undertaken in this paper. In section 3, we outline the bases of reflective equilibrium as a theory about epistemic justification of moral judgement in four steps. Throughout the rest of the paper, we develop a framework for analysing the relevance and different roles that empirical studies may reasonably have in these four steps, when applied to principles for priority setting. More specifically, in section 4, we introduce our framework. In section 5, we focus on studies that are relevant to identify various kinds of moral views. In section 6, we discuss studies that are relevant to identify moral principles. In section 7, we discuss a number of studies that are relevant to identify how the so-called equilibrium process unfolds. In section 8, we discuss studies that are relevant when the process of moral reasoning goes from the individual to the collective level. In section 9, we sum up and conclude.

## Preliminaries

Healthcare priority setting is an essentially interdisciplinary field of inquiry. While several scholars in the field have been working on mid-level moral principles to answer how resources should be allocated, others have been conducting empirical research on public views about this question. We focus on the ways in which this field of empirical research may be relevant for the justification of mid-level moral principles guiding priority setting.

Furthermore, we will assume that the following discussion takes place in a publicly-funded healthcare system and that the argument applies to priority setting on a group as well as an individual level.

### Mid-level moral principles

Ethical principles are characterised at both high-level and mid-level.^29 30^ High-level ethical principles such as utilitarianism or deontological views aim to provide general answers regarding what should be done and why. But to formulate mid-level ethical principles rather rests on the assumption that it is useful and reasonable that decisions are guided by principles that are applicable in specific areas, in this case allocation of healthcare resources. To characterise such mid-level principles is not to copy high-level theories and paste them into just any context. High-level theories are rather part of one’s toolbox in that project. To develop such principles on a mid-level, involves normative judgements regarding priority setting which, implicitly or explicitly, are based on ethical theory.

### Content and process studies

We focus on the empirical field aiming to explore public values on priority setting. The terminology is notoriously ambiguous in this field, for example, the terms ‘public values’, ‘public views’, ‘societal preferences’, ‘social values’ and ‘public attitudes’ are used to denote the data collected in these studies. We shall, following Baker *et al*,^5^ use ‘public values’ to refer to values held by an individual or group, more specifically, the values stated by informants in the empirical studies cited in this paper. While there are several such studies exploring values held by different stakeholders, for example, caregivers and patients, we primarily focus on the views held by the public.^5^,^12^

While these studies primarily are concerned with the *content* of public values, we shall also draw on a closely related field about the *process* according to which people arrive at their moral judgements.^31^,^40^ The latter should not be confused with procedural justice which is concerned with the process according to which decision-makers arrive at a certain *decision* regarding distribution. Rather, we are concerned with the process of moral reasoning and how one, according to this process, arrives at moral judgements. Accordingly, we will use a diverse set of empirical studies to illustrate this process.^2^ Some of these studies may come to be overturned by future research as is characteristic of the scientific process. Rather than overturning this model of moral justification, this would further emphasise the importance of taking empirical work seriously when striving for moral justification.

In section 4 and onwards, we discuss the ways in which empirical research of these kinds may be relevant for the justification of mid-level moral principles. Before we turn to the discussion of relevance, we shall spell out the basis of the Rawlsian framework reflective equilibrium in the next section.

## The notion of reflective equilibrium

Reflective equilibrium is primarily a theory about what makes moral judgements epistemically justified; however, it is often related to as a method for moral reasoning.^3^ 24,2841 42 Moreover, it is sometimes used to denote the goal or endpoint of such a process, namely where moral judgements cohere (and then, supposedly, are justified). Since it is doubtful that one ever reaches such an ideal state, as also acknowledged by Rawls,^25^ it is more appropriate to think about the goal of the process of moral reasoning as arriving at ‘resting points’ rather than ‘end-points’. In the following, we shall primarily relate to reflective equilibrium as a theory about epistemic justification of the moral judgements in a given resting point. That being said, any such theory will be closely related to the method of moral reasoning according to which one arrives at that resting point.

The process of reflective equilibrium is a working back and forth between: (a) one’s considered moral judgements about particular cases, and (b) ethical principles. In the following, we shall illustrate the process by dividing it into four separate steps. We primarily structure the process in these separate steps for clarificatory reasons, in practice the steps will often interplay.

### Identifying and filtering judgements—first step

The first step may be described in the words of Tersman^27^: it ‘…begins with filtering one’s moral views so that one is left only with those qualifying as considered judgements…’ (p. 47).^4^ For a judgement to qualify as a considered, rather than a raw unreflected moral judgement, it should meet a number of requirements. It should be of the kind in which one has strong faith, that one has the relevant information about and it should not be best explained by one’s self-interest or prejudices (more on such filtering below).

### Formulating principles—second step

The second step is to formulate ethical principles that can account for the considered judgements. These principles may vary in their degree of specificity,^26^ they may be of the general kind such as ‘euthanasia is wrong’ or more specified such as ‘euthanasia is wrong when offered to patients who are not terminally ill’. Note that the considered moral judgements may also vary in their degree of specificity. Accordingly, there is no clear-cut line between what qualifies as a considered moral judgement and what qualifies as an ethical principle.

### Working back and forth—third step

The third step involves a working back and forth, sometimes modifying the considered judgements and sometimes modifying the ethical principles, evaluating and revising each category on the basis of the other. It is important to stress that both categories are always revisable in the sense that neither considered judgements nor ethical principles are immune to revision. The process strives towards coherence, and at any given resting point, the judgements within a given set are epistemologically justified in virtue of them being coherent. This makes reflective equilibrium a coherentist view of epistemological justification of moral judgements.^27^

Adherents of coherence theory tend to agree that logical consistency among a set of judgements is a necessary but not sufficient condition for coherence. To increase the justificatory force of a set, one should also strive to ensure that members of a set are mutually supportive and explanatory.^24^,^28^ Since such support and explanatory power reasonably come in matters of degree it seems right to assume that it is not the case that some moral judgements are justified and some are not; rather, moral justification is a matter of degree, see also Tersman.^27^

There is a general tendency among coherence theorists to believe that large justificatory circles are better than small ones. In relation to moral reasoning this is often referred to as wide (as opposed to a narrow) reflective equilibrium.^24^,^28^ At the individual level there are at least two ways in which one may extend this process to increase the justificatory weight of the resting point.^5^

First, one may strive towards an extension of the content. This involves what Daniels refers to as background theories. According to Daniels^24^ reflective equilibrium: ‘…is an attempt to produce coherence in an ordered triple of sets of beliefs held by a particular person, namely, (a) a set of considered moral judgements, (b) a set of moral principles and (c) a set of relevant background theories’ (p. 258). Hence, a process involving (a) and (b) would be a narrow reflective equilibrium while including (c) would be a wide reflective equilibrium, on this view. One may ask what work (c) is supposed to do here. Daniels^24^ says that ‘[t]he background theories in (c) should show that the moral principles in (b) are more acceptable than alternative principles on grounds to some degree independent of (b)’s match with relevant considered moral judgements in (a)’ (p. 259). For example, as we shall discuss below, Rawls’s theory of stability assumes a particular theory of our psychology which implies that only moral principles that are consistent with our tendency to reciprocity can be correct.^6^

Second, one may strive towards an extension of the process in terms of alternative views. The more alternative views one considers, before one arrives at a given resting point, the more justificatory force that resting point has. This means that a position should be tested against other relevant moral positions. Note that this extension does not refer to what is included in a given resting point but to the process according to which one arrives at the resting point.

There is a further way in which reflective equilibrium can be expanded. However, this third way is wide in a somewhat different sense than the two outlined above, namely that it involves more than one individual. Accordingly, reflective equilibrium can be thought of as both an individual and a collective approach to moral judgement. We discuss this extension as a fourth step below.

### Social equilibrium—fourth step

The fourth step takes place on a public level. The goal with this step is to achieve coherence between the judgements held as justified by the different people in a society. For instance, there needs to be some agreement in a society of principles of justice. Rawls characterises this as wide and general reflective equilibrium.^43^ This is a goal that is achieved by collective deliberation. In a just society citizens must be able to voluntarily agree to the principles that organise their cooperation. How can a group come to a shared agreement on such norms? We extend justification by talking to more people, and, in particular, by more people sharing our judgements for justifiable reasons.

## A model of reflective equilibrium and empirical studies

As outlined above, there are four steps of reflective equilibrium: (1) identifying and filtering moral judgements, (2) formulating principles, (3) working back and forth between (1) and (2) and (4) from the individual to the social. There are several important upshots for empirical studies of moral judgements and their implications for the justification of mid-level moral principles. As reflective equilibrium is a *process* that aims to reach justification for the *content* of normative judgements we will, in the following, discuss these implications and focus the discussion on these two aspects of the empirical study of moral judgement. The distinction between the *content* of moral judgements and the *process* according to which people arrive at those judgements with the four steps of reflective equilibrium, give us a matrix of eight positions (table 1). The table is supposed to give the reader an idea about how the discussion in the subsequent sections will unfold. The matrix exemplifies the kinds of empirical issues that are relevant for reflective equilibrium, and consequently for the processes of arriving at justified principles for priority setting. In the following sections, we will outline the content and reasoning behind these examples.

**Table 1:** The relevance and roles of empirical studies

	The content of normative judgement	The process of arriving at normative judgement
Identifying and filtering judgements	Identify considered judgements.	Identify if, and if so how, and with what our considered judgements vary.
Formulating principles	Identify ethical principles.	Identify our responses to principles.
The equilibrium process	Identify how we evaluate judgements and principles.	Identify methods, alternatives and mistakes of reasoning.
Social equilibrium	Identify socially acceptable principles.	Identify the preconditions for social agreements.

## Identifying and filtering judgments—first step

### Identifying judgements

Chan *et al*^2^ surveyed a sample of the UK public regarding which features they thought were morally relevant for ventilator triage. Informants were asked what relevance they ascribed to 30 different features for triage. For example, age, recovery prospect, societal contribution, gender, career, infection responsibility, economic status and criminal history. The study contains a sophisticated analysis of the strength of people’s views about these features. However, the extent to which the views collected were considered rather than non-considered in the Rawlsian sense is not clear. At least, no measures to collect the former rather than the latter were undertaken. To what extent such filtering is possible may, of course, be discussed. The working hypothesis for this paper is that moral judgements may be more or less considered. Traditionally, the filtering of moral views has often been thought of as being concerned with the views of the armchair philosopher. However, empirical studies may also be designed to uncover informants’ considered (rather than non-considered) judgements. There are several procedures that can be undertaken to strive for this purpose. In the following we discuss examples of such procedures.

### Filtering judgements à la Anderson: the circumstances of reasoning

For judgements to qualify as considered judgements, they should not be best explained by one’s prejudices or by self-interest. One strategy to avoid such biases is discussed by Anderson^33^:

‘We can empirically investigate whether certain biases—thought tendencies that we have reason to reject for purposes of adjudicating moral diﬀerences—have distorted our moral thinking. Such investigation may also discover methods for blocking or counteracting these biases. Implementation of these methods may then yield diﬀerent beliefs, which are more trustworthy for avoiding the biases in question’ (p. 23).

Anderson uses historical case studies regarding the abolition of slavery to investigate the issue of moral deliberation. In particular, she shows how avoiding biases is very difficult if the process of justification is conducted without input from different social perspectives. Whereas she investigates moral progress regarding slavery in particular, we apply her ideas in a more general way to allow them to be applied to priority setting ethics.

For example, some socioeconomic groups are more powerful than others. Against the background of Anderson’s ideas, researchers can avoid biases in moral judgement by using methods that inform the powerful groups about the needs and interests of the less powerful groups. This may be a way to avoid moral judgements that are best explained by self-interest and prejudice. Furthermore, Anderson’s ideas seem to have a more simple and straightforward implication, namely that researchers should strive towards including informants in their studies being from different socioeconomic groups. That is, securing input from different social perspectives.

### Filtering judgements à la Fishkin

One concrete method for collecting considered judgements is through deliberative opinion polls. Fishkin^38^ takes deliberative opinion polls to be designed to answer the question of ‘[w]hat *would* the public think if its members were effectively motivated to become more thoughtful and informed?’ (p. 161. italics in original). He sets out to solve three problems with standard polling: (a) the public may not be informed, (b) the public may not be clear on the relation between different judgements and (c) the public may not be motivated to form judgements on the topic asked about.

With regard to priority setting, the public may, for example, be uninformed about the size of the healthcare budget or the costs and benefits of different interventions. The public may also not be aware of, or considerate about, the alternative use of resources. That is, when health intervention *A* is publicly funded this implies that intervention *B* cannot be funded. Moreover, people may have no real views on a topic but still express judgements on that topic. There are cases where large parts of the public have expressed support or resistance to policies that do not exist and for some issues the distribution of views is best explained by random variation.^38^ This is not to say that people are incapable to form considered judgements but rather shows that some preconditions must be in place for the development of considered judgements.

During deliberative opinion polls the participants gather for a long weekend of deliberation on some policy issue. Before the meeting, they are surveyed on their views and then briefed on the topic they are to discuss. This briefing includes arguments for and against the competing policy suggestions. During the weekend of deliberation, the participants are assigned randomly to small groups that are led by moderators tasked to guide the debate on the issues. After the weekend the participants again fill out the initial survey. The result of this process of proper deliberation based on the facts of the matter is that people’s judgements change. In particular, they get more informed, more capable of applying ideological frameworks and their views stop fluctuating at random.^38^ People form considered judgements. However, although deliberative opinion polls can help with making judgements more considered, there are other challenges with such polls. For example, there may be a selection problem because of small numbers which may give rise to bias and there may be only specific groups in society that can spare a ‘long weekend’ for deliberating policy issues.

### The role of empirical studies

Structuring empirical studies against the background of these considerations may avoid the biases of prejudice and self-interest which allow researchers to come one step closer to considered judgements. Of course, these efforts may be done to different degrees which, in turn, reasonably result in different degrees to which studies can capture considered rather than non-considered judgements. As have been argued by Kahane,^44^ to the extent public values are considered (rather than non-considered) there are no reasons to ascribe more (or less) weight to public values as compared with armchair intuition. Savulescu *et al*^22^ argue in a similar vein when sketching their proposal about how empirical studies of public values are relevant for moral justification. However, they say something stronger, namely that ‘…moral intuitions…*can* confer justification on moral views and, by extension, on policies that implement those views—*if* these intuitions meet certain conditions…’ (italics in original, p. 656). At a general level, their view accords with the approach to reflective equilibrium proposed in this article. However, they seem to rely on a distinction which cannot be formulated within our approach, namely that there are two distinct sources of moral justification: moral theory and attendant armchair intuitions on one side, and public values on the other. In the framework that we propose, moral justification comes from reflective equilibrium and the input to that process is, and should be, both moral theory and public values. Furthermore, Savulescu *et al* primarily focus on the importance of collecting the public’s considered rather than non-considered judgements, and what weight such judgements should have compared with armchair considered judgements. The framework that we propose also accounts for the extent to which judgements are considered but it also shows that there are several other ways in which empirical studies of public values can be relevant to moral justification. This approach will be further clarified in the coming sections.

## Formulating principles—second step

The next step of reflective equilibrium is to formulate principles that can account for considered judgements. This is also a stage where empirical studies are important.

### The example of Otsuka and Voorhoeve: how to choose among principles

Consider the following case. Otsuka and Voorhoeve^45^ draw on empirical studies when sketching an argument for egalitarianism rather than prioritarianism when it comes to principles about distributive justice.^7^ The argument targets the moral motivation for why it matters more to benefit the worse off. According to egalitarianism, it is bad in itself if someone is worse off than someone else. This makes egalitarianism a *comparative* view about justice. According to prioritarianism, it matters more to benefit a person the worse off that person is. This means that prioritarianism is concerned with people’s *absolute* levels of welfare.^46 47^

Otsuka and Voorhoeve^45^ refer to several empirical studies^8 16^ and adopt a health description in terms of mobility^15^: (1) full health, (2) slightly impaired, (3) severely impaired and (4) very severely impaired. They point out that respondents express different importance to people being worse off dependent on whether there are other people to be helped. Consider the following two cases.

Intrapersonal case: Jack learns that he has a 50% chance to develop either a condition A which would make him slightly impaired and a 50% chance to develop a condition B which would make him very severely impaired. There are preventative pharmaceuticals for both A and B, but they must be taken before Jack knows if he in fact will develop A or B, he cannot take both, and the drug for A is ineffective to B and vice versa. If Jack is given the drug targeting A and in fact develops A he will be in a state of full health. However, if he is given the drug targeting A and in fact develops B he will be very severely impaired. If Jack is given the drug targeting B and in fact develops B he will avoid very severe impairment but be severely impaired. However, if he is given the drug targeting B and in fact develops A he will be slightly impaired.

Interpersonal case: There is an even number of healthy people. Half of these people will develop A which makes them slightly impaired, and the other half will develop B which makes them very severely impaired. In this case the respondent is told that he or she knows which individuals will develop which condition. However, only the individuals in one of the two groups can be provided with their drug. Therefore, a choice needs to be made.

The studies to which Otsuka and Voorhoeve refer suggest that in *the intrapersonal case* respondents are indifferent to whether Jack is given the drug that targets *A* or the drug that targets *B*. However, in *the interpersonal case* they are not, rather there is a strong tendency to prefer treating the group in which individuals will develop *B* rather than the group that will develop *A*. Accordingly, there seems to be a shift in moral judgement when we move from *the intrapersonal case* to *the interpersonal case*. Otsuka and Voorhoeve conclude that the relevant considered judgement therefore is that what matters morally is that some are worse off than others (comparative claim) and argue that since prioritarianism cannot account for this shift and egalitarianism can this provides an argument for the latter rather than the former.^8^ This indicates how empirical research can help us formulate the principle that best accounts for the considered judgement.

### Identifying responses: how we react to principles

At this stage, empirical information regarding our reasoning about principles and the circumstances under which we accept principles may also become important. This makes research on reciprocity, such as the experiments conducted by Fehr and Schmidt,^37^ Konow^40^ and Cappelen *et al*^34 35^ important for the question of the acceptability of principles. Their results indicate that we tend to act in a manner consistent with a principle of fairness which includes a concern for reciprocity. Theories in this field tend to be formulated in the terminology of economic theory. Such theories, then, say that an individual’s utility function has a form that implies that the utility an individual gets from some resource is a function of both the value afforded by this resource to the individual and of the distribution of the resource. There is disutility in non-reciprocal distributions and hence both from getting less and more than what would be reciprocal. The utility that a person would get out of a specific allocation in, say, the ultimatum game will be a function of how much he or she receives of what he or she finds valuable and how much the actual allocation diverges from the person’s ideal allocation, which may take both equality and responsibility into account. To maximise utility, it is therefore not enough to gain as much advantage as possible, fairness is also important.

This indicates that empirical work on principles is relevant for the acceptability of normative principles. Formulating principles that are unlikely to be accepted in practice are unlikely to be the result of a reflective equilibrium.

## Going back and forth—third step

### Evaluating categories

One sense in which categories are already evaluated in step 2 was illustrated by Otsuka and Voorhoeve’s argument above. Here empirical studies provided the process of reflective equilibrium with moral judgements about particular cases that may, in a second step, be accounted for by formulating moral principles. An underlying idea here is that the plausibility of a given moral principle can be assessed based on the extent to which this principle can account for a considered moral judgement.

Consider next Daniels’^24^ account of wide reflective equilibrium according to which it concerns the aim of producing an order triplet of considered judgements, moral principles and background theories. If this is true, however, a resting point will not only express moral principles and judgements, but also background theories. In turn, this means that just looking at public values about some particular case risks being misleading if the results are used in a situation where the background theories do not hold. For instance, if we take moral principles from a context where the facts make a case for holding people responsible (eg, well informed and competent patients involved in shared decision-making)^48^ and use them in another context where the facts do not allow holding people responsible (eg, priorities regarding healthcare for young children) then we will use empirically informed principles that do not apply to the case at hand. Accordingly, a given background theory must be considered in a context where that background theory holds in order to be relevant for justification.

Dworkin’s^36^ theory of interpretation and normative deliberation could also serve as an example here. For Dworkin, deliberation concerns developing the best possible interpretation of, for example, an institution. To proceed with such a project, clear information regarding the history, function and practices of an institution is necessary. To know such things, we need to know how people understand and evaluate the institution at hand. An interpretation that did not take such things into account would be impoverished and probably misleading. Studies in the healthcare context can shed light on this part of the reflective equilibrium process. For example, Svantesson *et al*^49^ use reflective equilibrium as a method for approaching moral issues in referrals to the intensive care unit. By conducting semi-structured interviews with clinicians and family members, they find that evaluating potential principles in a structured way can facilitate agreement on principles and that the process also brings to the fore relevant implications for how to organise healthcare provision. Empirical information about how people evaluate the institutions that deliver healthcare can enrich the process of reflective equilibrium striving towards justified principles for priority setting.

### Methods, alternatives, mistakes: how we go back and forth

In general, the process of reflective equilibrium consists in going back and forth between particular judgements and principles. This process will turn on how we evaluate reasons for different views. This means that aspects of our reasoning abilities will be relevant at this stage. As we have seen, it is quite a complex undertaking to strive towards a reflective equilibrium including weighing and making trade-offs between different values. This is a process that may go wrong if we make mistakes in terms of reasoning of the kind outlined by Kahneman.^39^ For example, if we are prone to confirmation bias—the fallacy of only looking for evidence that supports our prior belief and discounting evidence that supports other views—this clearly undermines the pursuit of a well-reasoned reflective equilibrium. Another tendency to which we seem to be prone, and that has particular salience when it comes to the evaluation of priorities is known as the endowment effect. This effect explains why people would evaluate the same thing differently based on whether it is in their possession or not. To give an example, the exact same coffee mug may be valued to, for instance, US$15 by a person when not owning it, but at US$20 if he or she owns it. That is, evaluation varies not only with the thing being evaluated but also with ownership. This seems a fallacy, at least in the context of priority setting. Understanding when deliberation may go wrong and under what circumstances it succeeds will improve the process of reflective equilibrium. This implies that empirical results regarding *how* we reason will be relevant to moral justification.

### Alternative views

The argument for widening reflective equilibrium in terms of alternative views is that the more relevant such views one considers the more justificatory force a given resting point has. Rawls suggests that since moral philosophers cannot, for practical reasons, take *all* moral views into account, they must settle with the second-best option which is to ‘…characterize the structures of the predominant conceptions familiar to us from the philosophical tradition, and to work out the further refinements of these that strike us as most promising’ (Rawls, p. 8).^26^ Rawls mentions rather than argues for this source of alternative views. It may, after all, seem quite reasonable since philosophical writings are sources where moral ideas are (ideally) well developed and clearly expressed.

A further source of alternative views may be empirical studies of public values. Studies of people’s moral views may provide the process of reflective equilibrium with alternative views which, in turn, may increase the justificatory force of a given resting point. If the role of these studies is to provide reflective equilibrium with alternative views, there seems to be clear implications for how these empirical studies should be designed. For example, questionnaire studies with predefined moral views, such as study by Chan *et al*,^2^ would not do the work required. The aim of such studies would be to provide alternative views that would ‘surprise’ the ethical analysis with views that may not come up in the armchair. Therefore, studies with more qualitative designs such as interviews or focus group studies are more promising to provide alternative views.^9^

## Expanding the process from the individual to the social—fourth step

### Public values as a tiebreaker

Consider a situation in priority setting where there is a tie between two allocations: *A* and *B*. It is a tie in the sense that there are no substantive reasons to choose *A* over *B* (or vice versa) and one cannot choose both. However, a decision needs to be made. One possibility is to break this tie by flipping a coin but one may argue that such ties should be broken by appealing to public values. This would be an alternative way to address the question of applying empirical studies to the justification in priority setting: in a case when there are no substantive reasons for choosing *A* rather than *B* (or vice versa) but there are public values in favour of *A* then *A* should be chosen. We believe that there are at least two reasons to be cautious about using public values to break ties in this way. First, if the interpretation between the reflective equilibrium process and public values sketched in this paper is correct, then public values will already have played a role in coming to the moral draw at hand. Using them again as a tiebreaker would risk double counting public values. Second, and more importantly, adherents of this position need to explain *why it matters* that one allocation is in line with public values. A tempting answer may be to refer to the value of democracy—it is valuable if social institutions are governed by the will of the people affected by these institutions, or something to this effect. While democracy may, indeed, be an important value one may ask why the value of democracy should be ascribed the modest role of a tiebreaker. We believe that there is something else lurking in the background when the tiebreaker argument is appealed to, namely that democracy does have a central role here but a more complex role than suggested by the tiebreaker argument. There are at least two ways that public values can serve as important factors on this social and democratic level which we will discuss below.

### The preconditions for social agreements: the case of stability

Finally, let us turn to the side of reasoning and the psychological aspects of social agreements. Here Rawls may serve as an example. His notion of wide general equilibrium rests on the assumption that citizens can be and are both reasonable and rational. Roughly, a person is reasonable if she can accept cooperation on fair terms and she is rational if she takes efficient measures to reach her goals. If this account of human psychology is correct and a precondition for social equilibrium, then empirical research on whether presumptive public values are consistent with peoples’ rationality and reasonableness will provide indispensable information for coming to an agreement on principles for priority setting. Furthermore, a somewhat overlooked notion in the contemporary discussion about justice, is what Rawls refers to as stability. According to Rawls, a plausible principle of justice should satisfy a condition of stability which means that such a principle should be able to generate its own voluntary support over time. For instance, Rawls argued that considerations of stability provide one reason for why his theory of justice is more plausible than utilitarianism (Rawls, p. 126–130).^43^

To the extent stability is a plausible condition of adequacy for principles of distributive justice, it will still be *one* aspect of such principles which must be weighed against other considerations about these principles. The same goes for principles for priority setting. A promising way to include considerations of stability in priority setting ethics would be to build on the work by Munthe *et al*.^50^ Their argument is that established principles for priority setting should be complemented with a sustainability principle that takes into account a healthcare system’s ability to continue to function over time without loss of value. Whereas Munthe *et al* discuss examples such as antibiotic resistance and drug shortages we believe that a stability component in a sustainability principle would be about a different set of resources. A principle that is not sufficiently widely accepted for generating its own voluntary support over time would fail to be recognised as legitimate by (a) citizens and thereby decrease their willingness to contribute to healthcare in the form of tax and (b) healthcare professionals and thereby decrease their willingness to work in healthcare. Accordingly, if principles for priority setting are not tempered by considerations of stability healthcare systems are less likely to continue to function well over time as they risk a lack of monetary resources as well as healthcare professionals. Considerations about stability seem to create a new role for empirical studies of public values, namely that these studies seem to be of utmost importance for judging to what extent a given mid-level principle for priority setting satisfies the condition of stability.

### Identifying socially acceptable principles: general reflective equilibrium

Going beyond individual justification, we come to what Rawls calls wide and general reflective equilibrium. This is the idea that political justification of policy should be held in general between citizens, and that there ought to be a social process where they come to agree on certain core aspects of a resting point, that is, overlapping consensus. This indicates that empirical work on how we come to an agreement on public values in society and under what circumstances people accept principles over time become relevant for deliberation. Consider for example Acemoglu and Robinson^31^ who investigate under what circumstances democracy comes into being and survives and per implication also what makes people reject liberal democracy. Their theory is that people will accept and maintain a democracy if there is a state that is strong enough to solve people’s problems and a civil society strong enough to ensure that the state does not grow overly strong in a way that could stifle the liberties associated with democracy. The social value of democracy is, in this way, dependent on the organisation of civil society and the state. This kind of knowledge is clearly relevant for what principles for priority setting that can be justified in the fourth step. Are the institutional and social preconditions for agreement in place? Is it plausible that a given principle can serve as a common norm given what we know about how people reason about health and healthcare? Empirically informed work on this aspect of public values seems important for the project of identifying socially acceptable principles for priority setting.

## Summing up and conclusion

We have discussed the ways in which empirical research about moral judgement may be relevant for the justification of mid-level moral principles guiding priority setting. As it turns out, research on public values is important for moral justification. However, this importance is displayed in a less direct manner than one might think. While public values cannot straightforwardly decide the question about moral justification, these values may have several different roles which, in turn, have several different implications for the justification of principles. The framework developed throughout this paper can disentangle these different ways and make explicit what role a given study may have for justification (and by implication support researchers in the process of designing studies to ask the right research question(s) given their specific aim). Table 2 illustrates this by positioning the different kinds of studies discussed in the article in the framework of reflective equilibrium as it was outlined above in table 1.

**Table 2:** The relevance and roles of empirical studies—summing up

	The content of normative judgement	The process of arriving at normative judgement
Identifying and filtering judgements	Chan *et al*^2^Grover *et al*^4^Stafinski *et al*^9^	Anderson^32 33^Fishkin^38^
Formulating principles	Shah^8^Nord *et al*^15 16^Otsuka and Voorhoeve^45^	Fehr and Schmidt^37^Konow^40^Cappelen *et al*^34 35^
The equilibrium process	Svantesson *et al*^49^ Dworkin^36^	Kahneman^39^
Social equilibrium	Rawls^25 43^Munthe *et al*^50^	Acemoglu and Robinson^31^

Accordingly, there is a lot to be learnt from the vast number of empirical studies of public values, performed during the pandemic and in other contexts, but to fruitfully use these results we must develop a clear view of the roles and relevance of these results for justification. Empirical results regarding public values cannot determine which principles for priority setting we ought to accept, but such research has important roles to play during each stage of arriving at justified mid-level principles for priority setting.

## Data Availability

No data are available.

## Footnotes

See also [22Savulescu et al, (pp. 654-–656)]^22^ about problems with these two polar positions.

While the present paper focuses on studies of the “‘content’” and “‘process’” about moral judgement we remain open to the idea that there may be a broader set of empirical studies of potential relevance for our discussion. For example, empirical studies about the nature of principles and judgeements.

In spelling out the basis of reflective equilibrium here we are relying quite heavily on the outline of that theory in Gustavsson [30]. ^30^

See also Scanlon^42^ [42] who sketches the process in much the same way. As far as we can see there is no reason why one cannot, first, start the process with an ethical principle and, second, revise the principle in a way that makes it account for considered judgeements. As we understand Tersman as well as Scanlon this is just how the process normally goes in practice, something strikes one as morally wrong and then one tries to account for why this may be so. Furthermore, Tersman describes moral judgements as beliefs which seems to presuppose a cognitivist (as opposed to non-cognitivist) view on the nature of moral judgements. In this paper we prefer to leave open the question whether moral judgements are best understood in terms of beliefs or some expressivist account.

Precisely which of the extensions discussed below that Rawls himself would endorse carries little importance for present purposes. For a discussion on this issue see Daniels and Tersman [24, 27]. ^24 27^

See also [27Tersman, (pp. 56-–68)]^27^ for an illuminating example of a background theory.

For a comprehensive discussion about their argument see study by Bognar [18].^18^

Bognar^18^ [18] argues that the shift in judgement is not due to actual differences in people’s moral judgements but rather a difference in how people’s preferences were measured in these studies. However, in the present paper the Otsuka &and Voorhoeve argument is used as a mere illustration on how empirical studies may be used in moral reasoning. Therefore, to what extent Bognar is right in his critic carries little importance for this paper.

For examples of such studies see study by Stafinski [9].^9^
